# Hysteresis Loops Design for Nanoporous Ferroelectrics

**DOI:** 10.3390/ma18153606

**Published:** 2025-07-31

**Authors:** Xuan Huang, Fengjuan Yang, Lifei Du, Jiong Wang, Yongfeng Liang, Pingping Wu

**Affiliations:** 1 The Higher Educational Key Laboratory for Flexible Manufacturing Equipment Integration of Fujian Province, Xiamen Institute of Technology, Xiamen 361021, China; xeniahuang@126.com; 2 Department of Materials Science and Engineering, Xiamen Institute of Technology, Xiamen 361021, China; 3 College of Materials Science and Engineering, Xi’an University of Science and Technology, Xi’an 710054, China; dulifei@xust.edu.cn; 4 State Key Laboratory of Powder Metallurgy, Central South University, Changsha 410083, China; wangjionga@csu.edu.cn; 5 State Key Laboratory for Advanced Metals and Materials, University of Science and Technology Beijing, Beijing 100083, China; liangyf@skl.ustb.edu.cn; 6 Department of Physics, Quanzhou Normal University, Donghai, Quanzhou 362000, China

**Keywords:** porous ferroelectrics, hysteresis loops, ferroelectric properties, phase field model

## Abstract

The design and adjustable properties of nanoporous materials are important for current and future technological applications, research, and development. In addition, nanoporous ferroelectric materials have the potential to achieve competitive ferroelectric, dielectric, and piezoelectric characteristics. In this work, using the phase-field model, we found that the shape of pores in barium titanite ceramics governs the formation of the ferroelectric domain structure and the switching hysteresis loop. A remanent polarization-coercive field (*P*_r_-*E*_c_) diagram is introduced to denote the shape of the hysteresis loops. We performed a fundamental study in understanding how the domain structures affect the properties of domain-engineered porous ferroelectrics. Simulation results show that the hysteresis loop of porous ferroelectrics can be designed by controlling the shape/orientation of the ellipse-shaped pores. Numerical simulations also verify that the dielectric/piezoelectric properties can be improved with artificially designed porous structures. These phase-field results may be useful in the development of highly performing lead-free ferroelectric/piezoelectric materials.

## 1. Introduction

Porous ferroelectrics for the multi-grained system are extensively studied for their potential applications in piezoelectric energy harvesting devices and lightweight ferroelectrics [1,2,3,4,5,6,7,8,9,10,11,12,13,14]. Recently, the nanoscale porous ferroelectrics observed in nanowire [15,16]/nano thin film [17,18,19,20] structure exhibit larger piezoelectric response with stress effect, which makes it possible for the development of a new generation of ferroelectric/piezoelectric devices. As the geometric confinements originate from the pore shapes and interfaces at the nanoscale, the coupling between the ferroelectric domain structure and the elastic field and/or electric fields around the pores is particularly interesting. Experimental works [9,21,22,23,24,25,26,27] demonstrate that ferroelectric properties can be enhanced by adjusting porosity, pore size, and pore alignment. Vorotyntsev et al. introduced nanoscale pores in the size range of 1–10 nm into ferroelectric thin films, resulting in improved performance due to the reduction of permittivity and mechanical stress relaxation [21]. Suzuki et al. noted that porous BaTiO_3_ films exhibit anisotropic compressive stress, which contributes to an enhancement in both ferroelectric and piezoelectric properties [22]. Stancu et al. found that the introduction of porosity alters the microstructure, leading to a decrease in the dielectric constant as porosity increases [23]. Using nanoengineering techniques, Billah et al. discovered that controlled pore wall thickness contributes to highly strained lattice structures, resulting in a significant piezoelectric response in (Ba, Ca) (Ti, Zr) O_3_ thin films [24]. Delimova et al. observed that an increase in porosity reduced the magnitude of polarization and the hysteresis area of the hysteresis loop, as well as causing a tilting of the loops and a reduction in their rectangularity [25]. Zhang et al. [10,26] and Roscow et al. [27] also point out that the interconnected ferroelectric phase with isolated pore channels (or 2-2 nacre-like structures) exhibits superior mechanical properties.

These experimental works show that it is possible to achieve a special hysteresis loop by controlling the porosity of ceramics and changing the shape of the pores in ferroelectrics, or to improve the ferroelectric/piezoelectric properties by adjusting the shape of the hysteresis loop at a certain porosity. However, systematic studies are absent, especially in experiments. Despite the great time cost and effort required for experimental work, the size and shape are rather difficult to control in the experiments. The ultimate purpose is the creation of porous ceramics with the enhancement of ferroelectricity at a certain porosity or less reduction of physical properties at a large porosity level. In this aspect, simulation works, including phase-field modeling [28,29,30,31,32,33,34,35,36] and finite element modeling [1,7,26,27,37], show advantages in predicting the hysteresis loops with various possible nanostructured porous ferroelectrics. Understanding the fundamentals of ferroelectric domains on the physical properties of hysteresis loops can help us recognize the mechanism of the enhancement of the properties and reveal the way to enhance porous ferroelectrics.

In this work, to denote the designed hysteresis loop, a new remanent polarization-coercive field map was introduced to represent the shape of the switching hysteresis. One can imagine the shape of the hysteresis loop at a point on the map and trace the point with the change in porosity/shape of the pores. From a minimum pore size (at a porosity of 1.2% with a pore radius of 8 nm) to a maximum pore size (at a porosity of 60.1% with a pore radius of 56 nm), we designed three paths to investigate how the pore shape and the domain structure affect the hysteresis loop. To improve the physical properties of the porous ceramics, we studied the mechanism of the pores with their elastic field around them, and three possible methods are suggested to design/control the hysteresis loop. Finally, the dielectric/piezoelectric properties of porous ferroelectrics are predicted through comprehensive consideration.

## 2. Simulation Method

To describe the domain structures for porous ferroelectrics, firstly, we should separate the ceramic phase and pore phase with the order parameter *η*(*r*). If *η*(*r*) = 1, position *r* is occupied by the ferroelectric phase; if *η*(*r*) = 0, it is occupied by the pore phase. In the ferroelectric phase, the ferroelectric domain structure is described by the distribution of local polarization *P*_i_(*r*). The total free energy of the ferroelectric system includes bulk chemical Landau energy, domain wall energy, electrostatic energy, and elastic energy, i.e.,

(1) $$F_{electric}=F_{land}+F_{wall}+F_{elec}+F_{elas}$$

The *F_land_* here can be represented in terms of eighth-order polynomials:

(2) $$F_{land}\left(P_{i}\right)=\int_{V}\left(\begin{array}{c} \alpha_{1}\left(P_{1}^{2}+P_{2}^{2}+P_{3}^{2}\right)+\alpha_{11}\left(P_{1}^{4}+P_{2}^{4}+P_{3}^{4}\right)\\ +\alpha_{12}\left(P_{1}^{2}P_{2}^{2}+P_{2}^{2}P_{3}^{2}+P_{1}^{2}P_{3}^{2}\right)+\alpha_{111}\left(P_{1}^{6}+P_{2}^{6}+P_{3}^{6}\right)\\ +\alpha_{112}\left[P_{1}^{2}\left(P_{2}^{4}+P_{3}^{4}\right)+P_{2}^{2}\left(P_{1}^{4}+P_{3}^{4}\right)+P_{3}^{2}\left(P_{1}^{4}+P_{2}^{4}\right)\right]\\ +\alpha_{123}P_{1}^{1}P_{2}^{2}P_{3}^{3}+\alpha_{1111}\left(P_{1}^{8}+P_{2}^{8}+P_{3}^{8}\right)\\ +\alpha_{1112}\left[P_{1}^{6}\left(P_{2}^{2}+P_{3}^{2}\right)+P_{2}^{6}\left(P_{1}^{2}+P_{3}^{2}\right)+P_{3}^{6}\left(P_{1}^{2}+P_{2}^{2}\right)\right]\\ +\alpha_{1122}\left(P_{1}^{4}P_{2}^{4}+P_{2}^{4}P_{3}^{4}+P_{1}^{4}P_{3}^{4}\right)\\ +\alpha_{1123}\left(P_{1}^{4}P_{2}^{2}P_{3}^{2}+P_{2}^{4}P_{1}^{2}P_{3}^{2}+P_{3}^{4}P_{2}^{2}P_{2}^{2}\right) \end{array}\right)d^{3}x,$$

where *α*_i_, *α*_ij_, *α*_ijk_, *α*_ijkl_ (i, j, k = 1, 2, 3) are the phenomenological Landau coefficients, *V* is the volume of the simulated system, and the volume of *d*^3^*x* = *dx*_1_*dx*_2_*dx*_3_.

The energy of domain walls can be expressed by:

(3) $$F_{wall}\left(\partial P_{i}/\partial x_{j}\right)=\int_{V}\left[\frac{1}{2}G_{ijkl}\frac{\partial P_{i}}{\partial x_{j}}\frac{\partial P_{k}}{\partial x_{l}}\right]d^{3}x$$

where the *G*_ijkl_ is the gradient energy coefficient and the *G*_ijkl_ = *G*_klij_.

The electrostatic energy can be written as:

(4) $$F_{elec}\left(P_{i},E_{i}\right)=-\int_{V}\left(\frac{1}{2}\varepsilon_{b}\varepsilon_{0}E_{i}^{2}+E_{i}P_{i}\right)d^{3}x,$$

where *E*_i_ represents the electric field component, which depends on the polarization distribution and the boundary conditions, *ε*_0_ is the vacuum permittivity, and *ε*_b_ is the background relative dielectric permittivity.

According to Khachaturyan’s elastic theory, the elastic energy *F_elas_* can be written as:

(5) $$F_{\mathrm{elas}}\left(P_{i},E_{i}\right)=\int_{V}\left(\frac{1}{2}C_{ijkl}e_{ij}e_{kl}\right)d^{3}x=\int_{V}\left(\frac{1}{2}C_{ijkl}\left(\varepsilon_{ij}-\varepsilon_{ij}^{0}\right)\left(\varepsilon_{kl}-\varepsilon_{kl}^{0}\right)\right)d^{3}x,$$

where *C*_ijkl_ is the elastic constant of the material. *ε*_ij_ and *ε*_ij_^0^ represent the total strain and stress-free strain, respectively. For the ferroelectric phase, the stress-free strain caused by the local polarization is given by

(6) $$\varepsilon_{\mathrm{ij}}^{0}=Q_{\mathrm{ijkl}}P_{k}P_{l},$$

where *Q*_ijkl_ is the electrostrictive coefficient. It is convenient to define the total strain in the ferroelectric body as the sum of homogeneous and heterogeneous strains:

(7) $$\varepsilon_{ij}=\bar{\varepsilon_{\mathrm{ij}}}+\delta \varepsilon_{ij}\left(r\right),$$

where the homogeneous strain is defined so that

(8) $$\int_{V}\delta \varepsilon_{ij}\left(r\right)d^{3}x=0.$$

The homogeneous strain is determined by the elastic boundary condition of the material, when the system is under a clamped boundary condition, i.e., the shape of the material is not allowed to deform

(9) $$\bar{\varepsilon_{\mathrm{ij}}}=0.$$

When the material is under a stress-free condition, the homogeneous strain is calculated as

(10) $$\bar{\varepsilon_{\mathrm{ij}}}=\frac{1}{V}\int_{V}\varepsilon_{ij}^{0}d^{3}x.$$

The total strain can be calculated by Equation (7); hence, the elastic energy can be expressed as

(11) $$F_{\mathrm{elas}}\left(P_{i},\varepsilon_{\mathrm{ij}}\right)=\int_{V}\left(\frac{1}{2}C_{\mathrm{ijkl}}\left(\bar{\varepsilon_{\mathrm{ij}}}+\delta \varepsilon_{\mathrm{ij}}\left(r\right)-Q_{\mathrm{ijkl}}P_{k}P_{l}\right)\left(\bar{\varepsilon_{\mathrm{kl}}}+\delta \varepsilon_{\mathrm{kl}}\left(r\right)-Q_{\mathrm{klij}}P_{i}P_{j}\right)\right)d^{3}x.$$

In the present work, for the case of a porous ferroelectric system, the total free energy includes the contributions of ferroelectric energy and the energy of the pore phase

(12) $$F_{total}=\eta \left(r\right)\cdot F_{electric}+\left(1-\eta \left(r\right)\right)\cdot F_{pores}.$$

For simplicity, we set *F_pores_* = 0, which means that the pore phase does not contribute to the total energy of the system. The energy of the porous system can be reduced to the product of ferroelectric energy and order parameter *η*.

With all the energetic contributions, the temporal and spatial evolution of the ferroelectric polarization distribution *P*_i_(*r*, *t*) and the order parameter of ceramic phase *η*(*r*, *t*) can be described by the time-dependent Ginzburg-Landau (TDGL) equations

(13) $$\frac{\partial P_{i}\left(r,t\right)}{\partial t}=-L_{p}\frac{\delta F_{total}}{\delta P_{i}\left(r,t\right)},\;(i=1,\;2,\;3)$$

(14) $$\frac{\partial \eta \left(r,t\right)}{\partial t}=-L_{\eta }\frac{\delta F_{total}}{\delta \eta \left(r,t\right)},\;(i=1,\;2,\;3)$$

where *t* is time, and *L*_p_ and *L_η_* are kinetic coefficients. In our current work, Equations (13) and (14) are efficiently solved by the semi-implicit Fourier spectral method simultaneously.

In this paper, Barium titanate (BTO) has been chosen for this study, as a popular lead-free material. BTO can supply high dielectric constant and high piezoelectric constant, and the ceramics can easily be sintered in the air. The corresponding coefficients for Landau polynomials are listed here [38]: *α*_1_ = 4.124(T − 388) × 10^5^, *α*_11_ = −2.097 × 10^8^, *α*_12_ = 7.974 × 10^8^, *α*_111_ = 1.294 × 10^9^, *α*_112_ = −1.905 × 10^9^, *α*_123_ = 2.500 × 10^9^, *α*_1111_ = 3.863 × 10^10^, *α*_1112_ = 2.529 × 10^10^, *α*_1122_ = 1.637 × 10^10^, *α*_1123_ = 1.367 × 10^10^. The elastic constants and the electrostriction coefficients for BTO are chosen to be *C*_11_ = 1.78 × 10^11^, *C*_12_ = 0.964 × 10^11^, and *C*_44_ = 1.22 × 10^11^. *Q*_11_ = 0.10, *Q*_12_ = −0.034, *Q*_44_ = 0.029, where *C*_ij_ and *Q*_ij_ are the Voigt notation for *C*_ijkl_ and *Q*_ijkl_. All the coefficients are in the SI unit and T in Kelvin. The reduced gradient coefficient G_11_ = 2.0, G_12_ = 0, G_44_ = G′_44_ = 1.0. In the simulation, we set grid spacing *l*_0_ = 1 × 10^−9^ m (1 nm), a_0_ = 0.371 × 10^8^ C^−2^m^2^N, and *P*_0_ = 0.26 C/m^2^ at room temperature. For 180-degree domain walls, the domain wall energy density of BaTiO_3_ is about 2 g_11_a_0_*l*_0_P_0_^2^~0.01 Nm^−1^. This value is consistent with the experimental result of 10 erg/cm^2^ reported by Merz [39].

## 3. Results

### 3.1. Hysteresis Loops of Porous Ferroelectrics and the P_r_-E_c_ Diagram

**Figure 1:**
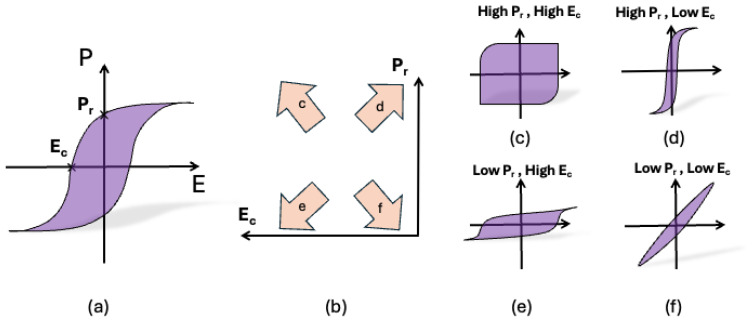
(**a**) Typical ferroelectric hysteresis loop with characteristic parameters of ferroelectric properties. (**b**) Schematics of the *P*_r_-*E*_c_ diagram with four typical types of hysteresis loops. (**c**–**f**) with different values of remanent polarization *P*_r_ or the coercive field *E*_c_.

To study the influence of the porosity and the shape of the pores on ferroelectric hysteresis loops, we would like to introduce a new coordinate diagram. As shown in Figure 1a, there are two key parameters in a hysteresis loop: the remanent polarization *P*_r_ and the coercive field *E*_c_. The remanent polarization *P*_r_ is the value of polarization at the external field return to zero, while the coercivity *E*_c_ is the reverse field required to reduce the polarization *P* to zero. For one special material, if the *P*_r_ and *E*_c_ are given, one can easily imagine the shape of the hysteresis loop. In this work, a *P*_r_*-E*_c_ diagram is presented with the *x*-axis variable of *E*_c_ and the *y*-axis variable of *P*_r_, as shown in Figure 1b. Figure 1b also illustrates four typical types of hysteresis loops. Hysteresis loops with high *P*_r_ and high *E*_c_ (Figure 1c) have great potential in applications of energy storage materials. Ferroelectric memory devices require high *P*_r_ to maintain the data signal, but small *E*_c_ to decrease the reverse electric field and increase the data density, as shown in Figure 1d. Applications for low *P*_r_ and high coercive fields are less reported, as they require a high field to reverse, but only output small polarization (Figure 1e). Ferroelectrics or dielectrics with linear hysteresis loops, as shown in Figure 1f, low *P*_r_, and low *E*_c_ are good materials for sensors.

It should also be noted that the shape of the hysteresis loop is not only dependent on the two parameters, but other factors, such as saturation polarization *P*_s_, can also significantly influence the hysteresis loop’s shape [2]. The rectangularity factor S = *P*_r_/*P*_s_ measures the degree to which the shape of a hysteresis loop is approximated by a rectangle [34]. Some anti-ferroelectric materials can perform constricted double-loop hysteresis [40], provide low *P*_r_ and low *E*_c_, and possess high energy storage density due to the anti-ferroelectric phase to ferroelectric phase transition [41]. Therefore, this *P*_r_-*E*_c_ diagram is specifically applicable to convex hysteresis loops. In general, the *P*_r_-*E*_c_ diagram is an interesting idea to illustrate the shape of the hysteresis loop for a certain porous ferroelectric. In this paper, we focus on a single-phase ferroelectric material (BaTiO_3_) with pores. Our study confirms that all the hysteresis loops we examined are convex.

**Figure 2:**
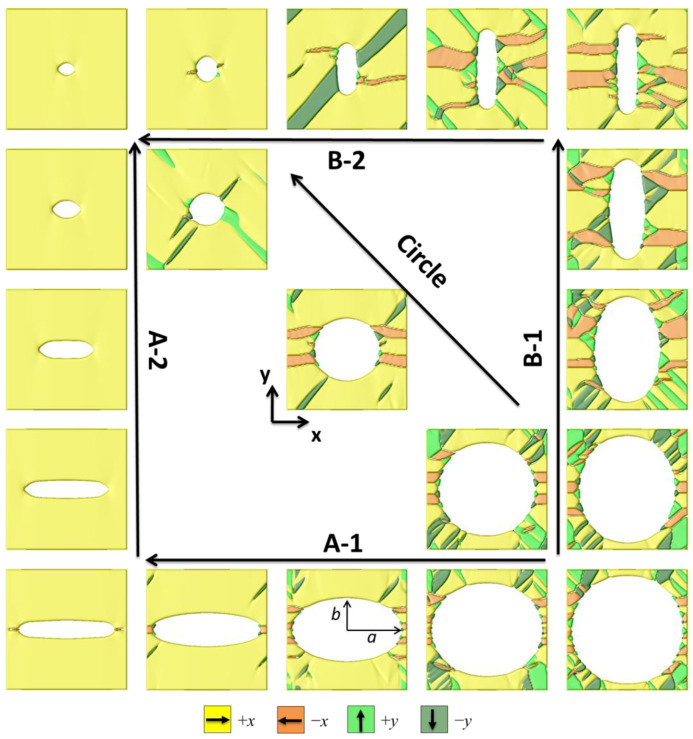
Ferroelectric domain structures at saturation with ellipse-shaped nanopores, path A: major axis along the *x*−direction; and path B: major axis along the *y*−direction. The size of the simulation is 128 × 128. The domains colored yellow, orange, green, and dark green represent +*x*, −*x*, +*y*, and −*y* ferroelectric domains, respectively.

In our current simulation, a simulation cell of 128Δ*x* × 128Δ*y* × 1Δ*z* discrete grid points is employed in this work, and the grid spacing of Δ*x* = Δ*y* = Δ*z* = 1.0 nm. Initial ferroelectric domain structures are generated by numerically solving TDGL Equations (13) and (14) with random seeding for initial polarization distribution. Periodic boundary conditions are employed in the *x* and *y* directions. The domain structures are evolved by 3000 steps to reach a stable state, then an electric field was first generally increased to saturation (~1.5–2.5*E*_0_) by a step of 0.05*E*_0_ along the *x*-axis, where *E*_0_ = 9.65 × 10^6^ V/m. From the saturation point, the electric field is reduced by reversal of field direction, and a second reversal of the field forms a complete hysteresis loop. We simulated a sequence of ferroelectric domain structures that form upon the decremental change of semiaxes of ellipse-shaped pores. The ferroelectric domains at saturation for porous ferroelectrics with various shapes of the pores, as performed by the phase field model, are shown in Figure 2. A sample domain structure with the largest pore size of 56 nm is demonstrated at the bottom right corner, and another example has the smallest pore size of 8 nm located at the left top corner of the diagram. We designed three paths to demonstrate ferroelectric domain structure evolution with pore sizes varying from 56 nm to 8 nm. Hereby, we denote *a* and *b* as the semiaxis of the ellipse pore along the *x* direction or the *y* direction.

As shown in Figure 2, we first reduce the characteristic length of semiaxis *b* (path A-1), then reduce the pore length along the direction of the applied field (path A-2). Conversely, along path B-1, the pore length *a* was reduced at first, then the pore length along the *y*-axis was reduced in the following path B-2. Path C decreases the length of the pore along the *x* and *y* axes simultaneously, i.e., reduces the radius of the circle-shaped pore. Among the simulated domain structures along the three paths, one can mention that the fractions of –*x* domains and *y* domains increase at saturation for large-size pores. This phenomenon can be explained by the strong depolarization field between two pore interfaces. Interestingly, with decreasing the pore length *a* (along path B-1), an increase in the size of all the four ± *x*/*y* domains is seen; however, while gradually reducing the semiaxis *b*, *x* domain expansion occurs and the volume fraction of −*x* and *y* domains is significantly reduced, as seen in path A-1 and B-2.

**Figure 3:**
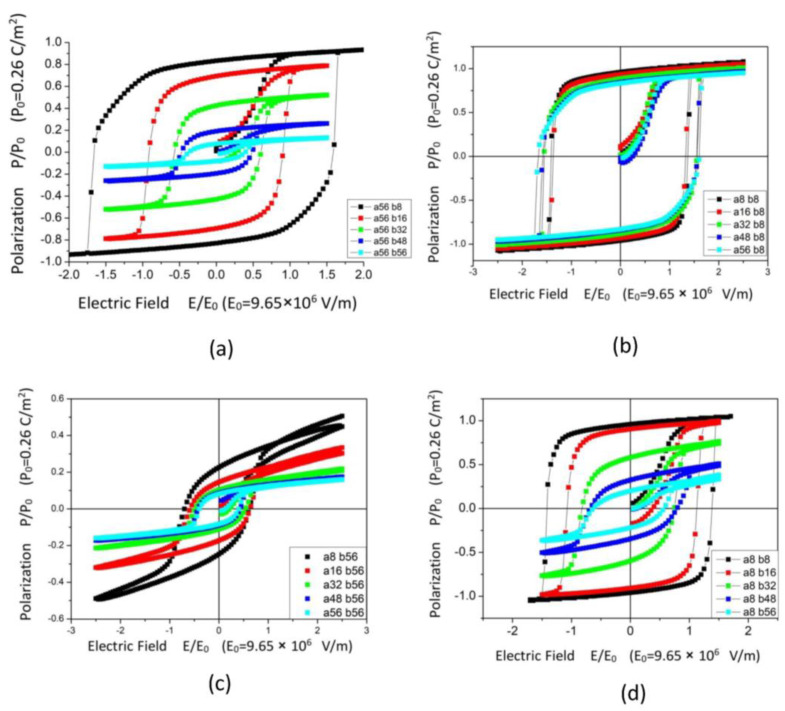
Simulated hysteresis loops along the path (**a**) A1, (**b**) A-2, (**c**) B-1, (**d**) B-2, where the parameters *a* and *b* denote the longest diameter along the *x* direction and *y* direction of the ellipse-shaped pore, respectively.

The hysteresis loops for ferroelectric structures along the paths A-1, A-2, B-1, and B-2 are shown in Figure 3a–d, respectively. The shape of the hysteresis loop can be strongly influenced by the porosity and the shape of the pores. Noticeable changes were found in Figure 3a (path A-1) and 3d (path B-2), where the *P*_r_ and *E*_c_ increase rapidly when decreasing the length of semiaxis *b* of the ellipse. In contrast, only small changes can be seen in the shape of the hysteresis loop in Figure 3b,c. Slight variations in the values of *P*_r_ and *E*_c_ were observed in the hysteresis loop.

In Figure 3a, as the length of the semiaxis *a* remains constant while the semiaxis *b* decreases from 56 nm to 8 nm, the shape of the hysteresis loop significantly changes. *P*_r_ increases nearly tenfold from 0.081*P*_0_ (*P*_0_ = 0.26 C/m^2^) to 0.833*P*_0_, and *E*_c_ increases from 0.4*E*_0_ (E_0_ = 9.65 × 10^6^ V/m) to 1.65*E*_0_, resulting in a square-shaped hysteresis loop. In the subsequent path A-2, as the *a*-axis length decreases from 56 nm to 8 nm, the shape of the hysteresis loop does not change significantly. *E*_c_ slightly decreases to 1.4*E*_0_, and *P*_r_ slightly increases to 0.95*P*_0_. Conversely, along the B-path, the changes in the hysteresis loop are different. In Figure 3c, with the *b*-axis length remaining constant and the *a*-axis length decreasing from 56 nm to 16 nm, there are minimal changes in *P*_r_ and *E*_c_. When the a-axis further decreases from 16 nm to 8 nm, *P*_r_ suddenly increases by 53%, from 0.15*P*_0_ to 0.23*P*_0_. In the following process, along path B-2, *E*_c_ dramatically increases to 1.40, and *P*_r_ increases about four times to 0.95*P*_0_, as shown in Figure 3d.

These changes can be explained by the domain structures shown in Figure 2. It can be observed that, along path A, the ellipse’s axes are parallel to the external electric field direction, and the porous BaTiO_3_ approaches full saturation. In contrast, along path B, the ellipse’s axes are perpendicular to the external field direction, the electrostatic effects result in numerous −*x* domains, which reduce the saturated polarization vector *P*_s_ and *P*_r_. Additionally, the presence of −*x* domains significantly lowers the nucleation difficulty of the polarization switching process and reduces the coercive field *E*_c_.

**Figure 4:**
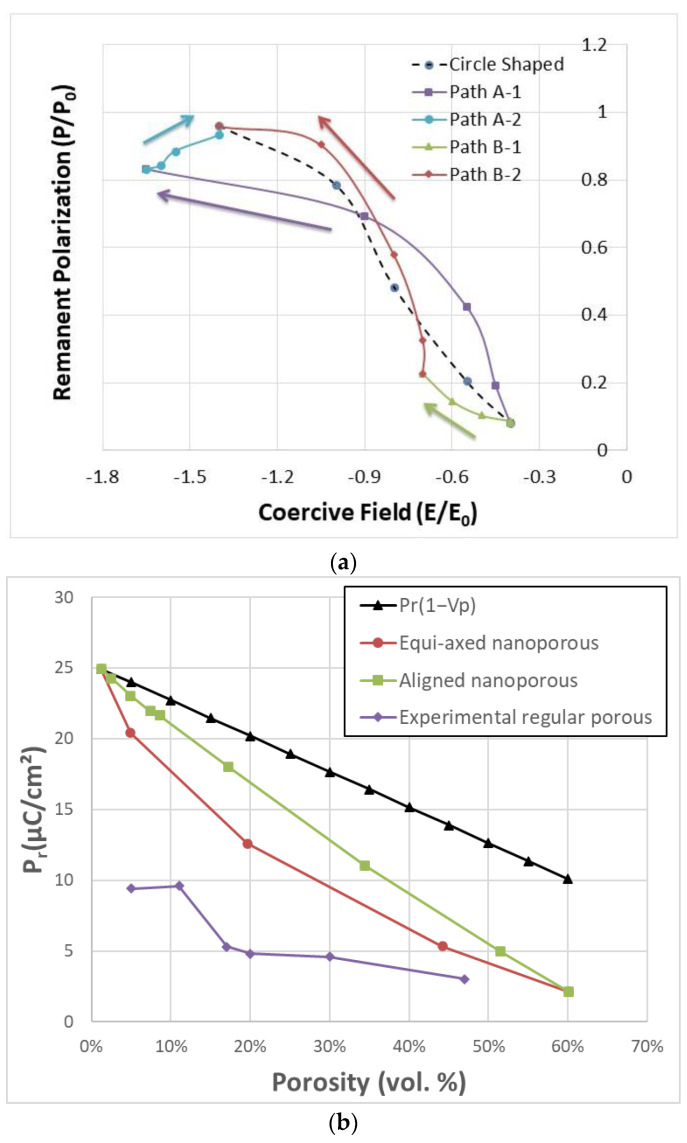
(**a**) Mapping remanent polarization and coercive field of the hysteresis loops of porous ferroelectrics in the *P*_r_-*E*_c_ diagram, arrows show the changes of *P*_r_/*E*_c_ along four paths A-1, A-2, B-1, and B-2. The dashed line represents the changes of *P*_r_/*E*_c_ along the path of the circle-shaped pores. (**b**) variation of remnant polarization with porosity in this work and experimental data.

Mapping remanent polarization and coercive field of the hysteresis loops of Figure 3 to the *P*_r_-*E*_c_ diagram suggested above made it possible to trace the changes in the shape of hysteresis with the pore size. As shown in Figure 4a, the starting point is located at the bottom right corner of the diagram with low *P*_r_/*E*_c_ and high porosity. Via path A or path B, the curve ended up at the top left point with large *P*_r_/*E*_c_ and low porosity, in which maximum *P*_r_ reached 0.25 C/m^2^ (0.959*P*_0_), and the maximum value of *E*_c_ is around 1.54 × 10^7^ V/m (1.60*E*_0_) at the point of *a* = 56 nm, *b* = 8 nm. By comparison, a path of circle-shaped pores is also illustrated in the diagram (dashed line in Figure 4a). Rapid changes in ferroelectric switching properties were observed along paths A-1 and B-2. The two paths share a common feature: the length of semiaxis *b* of the ellipse-shaped pore, which lies perpendicular to the applied field, is reduced.

For comparison with experimental works, Figure 4b shows the variation of *P*_r_ with porosity for circle-shaped pores, i.e., equi-axed porosity and aligned porosity (along the path A). With increasing porosity, the *P*_r_ for equi-axed porosity decreases more quickly than that for aligned porosity. Both curves are all lower than the ideal porosity model *P*_r_ = *P*_r_^0^ × (1 − V*_f_*), where *P*_r_^0^ is the remanent polarization of the dense material and V*_f_* is the volume fraction of porosity. These simulation results are in good agreement with experimental results in [8]. It should be noted that the predicted *P*_r_ values for nanoporous BaTiO_3_ are much higher than those observed in regular porous BaTiO_3_ experiments [42]. The calculated coercive fields in this work (~100 kV/cm) are significantly larger than those observed in experiments (~10 kV/cm in [42]) by approximately one order of magnitude. However, our computed values are consistent with those reported for other nanoscale BTO thin films [43], which exhibit coercive fields around 250 kV/cm.

**Figure 5:**
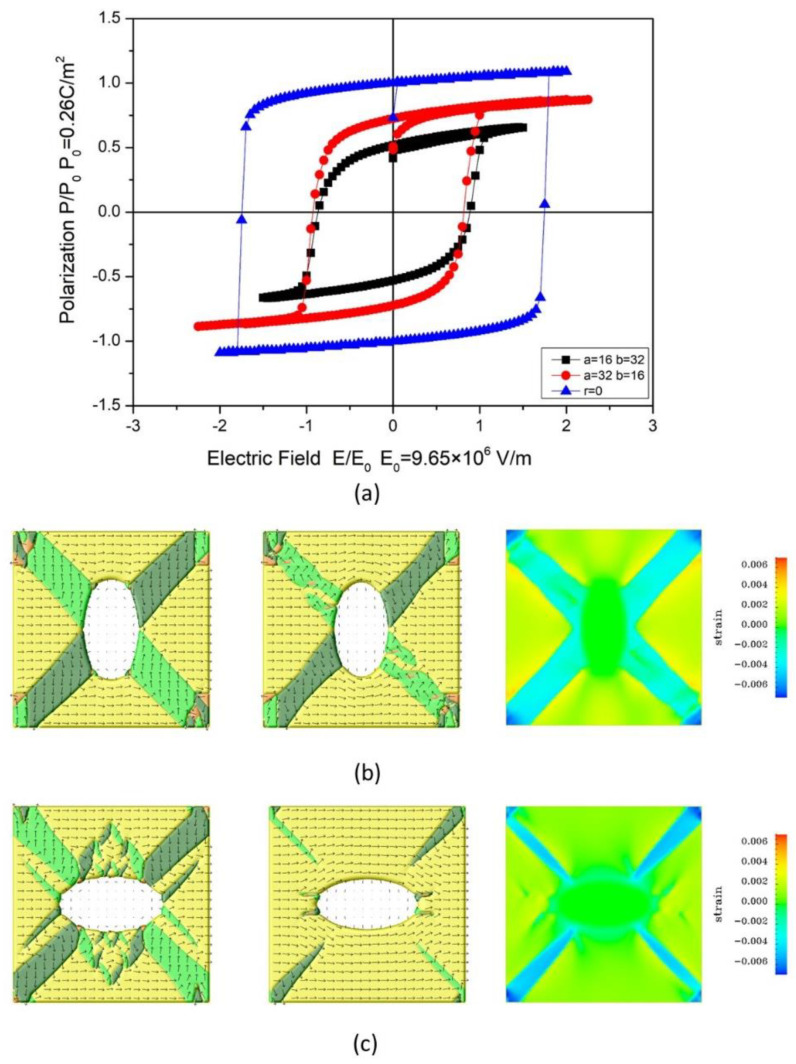
(**a**) Hysteresis loops for BaTiO_3_ with ellipse-shaped pores (*a* = 16 nm, *b* = 32 nm), (*a* = 32 nm, *b* = 16 nm), and comparison of dense ferroelectric. (**b**,**c**) illustrated the ferroelectric domain structures for ellipse shape (*a* = 16 nm, *b* = 32 nm) and (*a* = 32 nm, *b* = 16nm) with polarization vector at zero fields, saturation, and the elastic field (e_11_) distribution at saturation. (from left to right).

One of the interests is the effect of the pore shape, i.e., the change in switching properties as the symmetry of the pore in a ferroelectric is reduced. We focus on two examples sharing the same porosity: ellipse-shaped pores with semiaxes *a* = 16 nm, *b* = 32 nm, and *a* = 32 nm, *b* = 16 nm. The hysteresis loops and corresponding domain structures for simulated BaTiO_3_ with ellipse-shaped pores are illustrated in Figure 5a–c. The main difference between the two structures is that the major axis of the pore is along or perpendicular to the electric field direction. Note that the introduction of pores into dense ferroelectrics significantly reduces the remanent polarization *P*_r_. The pores supply nucleation cores, reduce the nucleation interface energy, and drive the motion of domain walls. This effect leads to the formation and growth of new domain configurations and results in a significant reduction in the remanent polarization (*P*_r_). The simulated results are also consistent with those reported in previous publications [44].

The domain structures evolved in 3000 steps to reach a stable state. As shown in Figure 5b, a stable stripe-like y-domain structure was observed in the porous ferroelectric, in which the long axis of the pore is perpendicular to the electric field. At saturation, a noticeable 90-degree domain wall motion can be seen, but the stripe-like y-domain structure is maintained under the combined action of strong electrostatic and elastic fields. On the other side, the porous ferroelectric body with its pores’ long axis parallel to the electric field generates a complex structure with many small fractional domains. These domains disappear under the saturated electric field. We also plotted the elastic field distribution of e_11_, which is also consistent with the corresponding ferroelectric domain structure. We believe that the combined action of elastic fields and electrostatic fields ultimately results in the remanent polarization of the structures, with pore shape perpendicular to the electric field direction being significantly lower than those with pores parallel to the electric field direction. This effect makes the remanent polarization even smaller than the circle-shaped structure at the same porosity level.

As we discussed above, the pores affect the ferroelectric properties of the material following the process below: First, an elastic field is generated around the pore, meanwhile, the pores also provide a new interface and lead to an electrostatic field, both of which contributes to form the ferroelectric domain structure, and the domain structure combined with the existing elastic field further determined final domain structure at saturation field. Consequently, the value of *P*_r_ and *E*_c_ of the hysteresis loop is strongly affected by the co-contribution of the elastic field and electrostatic field introduced by pores. It also should be noted that, in our previous study, we used random seeding for initial polarization distribution, reducing the volume fraction of residual −*x* domains between the pores can further enhance the remanent polarization of porous ferroelectrics.

### 3.2. Design the Hysteresis Loops

In this paper, we suggest three possible ways to design hysteresis loops for porous ferroelectrics. First is controlling the shape of the pores, i.e., the semiaxis *a* and *b* of the ellipse-shaped pores, which can efficiently change the ferroelectric hysteresis loop. The second is rotating the ellipse pore or the direction of the electric field, and adjusting the pore angle between the elongated pores and the poling direction. Third is trying to arrange the pore-making agents along one special direction to make the circle-shaped pores connected and create a “channel” along this direction, then controlling the direction of the applied field to achieve the loops required.

**Figure 6:**
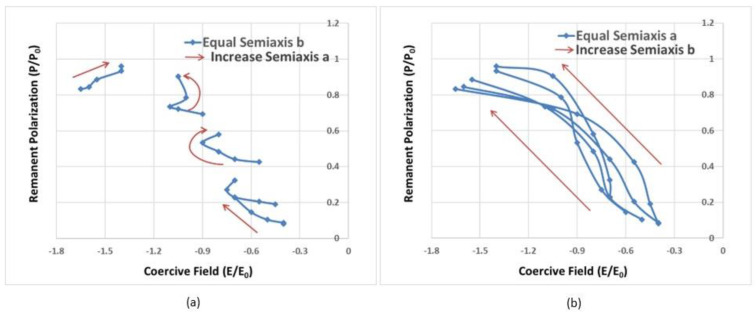
Controlling the shape of the ellipse pore with trend lines in the *P*_r_*-E*_c_ map: (**a**) increase the length of semiaxis *a* while keeping the length of semiaxis *b* unchanged. (**b**) Increase the length of semiaxis *b* while keeping the length of semiaxis *a* unchanged.

According to the simulation results above, one can note that the *P*_r_ and *E*_c_ are very sensitive to the length of the semiaxis *b* of the pore when the electric field is applied along the *x* direction. To further verify that assumption, we plotted two sets of trend lines in the *P*_r_-*E*_c_ map: one set increases the length of semiaxis *a* while keeping the length of semiaxis *b* unchanged. As shown in Figure 6a, as the length of semiaxis *a* plays a minor role in the hysteresis loop, mostly the remanent polarizations *P*_r_ upshift and the coercive field *E*_c_ change little with increasing the length of semiaxis *a*. However, if we held the length of semiaxis *a* as a constant and increased the length of semiaxis *b*, the value of *P*_r_ and *E*_c_ drastically enhanced (Figure 6b). This provides a possible method for designing the hysteresis loop: First, determine the *P*_r_ and *E*_c_ of the target hysteresis loop, then approximately adjust the shape of the pore based on the two sets of trend lines.

It is not easy to control the shape of the ellipse pore in practical experiments. As shown in Figure 2, one can notice that the pore structures along path A and path B show axial symmetry along the diagonal axis. Rotating the ellipse pore from 90° (perpendicular to the field) to 0° (perpendicular to the field) can greatly enhance the ferroelectric properties of the sample without changing the porosity. Rotating the direction of the electric field can also achieve the same results.

**Figure 7:**
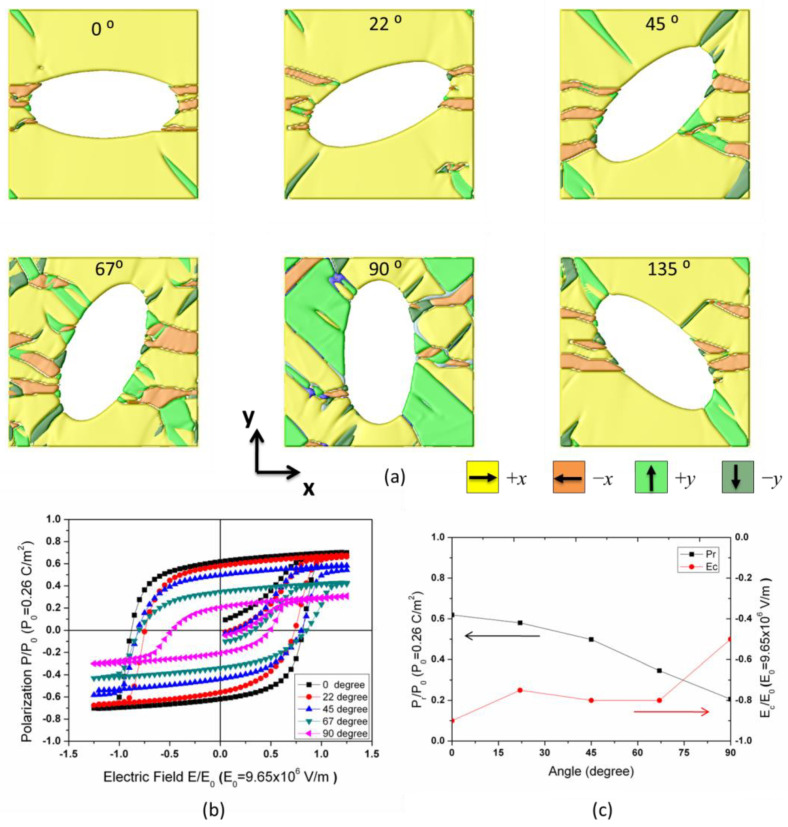
(**a**) Simulated ferroelectric domain structure for porous BTO with ellipse-shaped pores aligned along different directions, the angle between the long axis of the ellipse and the direction of the applied field is 0°, 22°, 45°, 67°, 90°, and 135°, respectively. (**b**) Simulated *P*-*E* hysteresis loops for porous ferroelectrics are plotted in (**a**). Note that the volume fraction of the pores remains the same. (**c**) The variation of remanent polarization and coercive field with the angle.

The ferroelectric domain structures of porous BaTiO_3_ with ellipse pore angles varying from 0 to 90° are shown in Figure 7a, and the polarizations are aligned under a saturation electric field along the *x* direction. Comparison of hysteresis loops variation with the domain structures is illustrated in Figure 7b. The formation of stripe-like *y* domains occurs and lowers both the *P*_s_ and *P*_r_ by increasing the pore angle, as shown in Figure 7c. A minor change in the coercive field was observed when the pore angle increased from 0 to 67°; however, if the elongated pores aligned perpendicular to the applied field, the coercive field drops rapidly with the broadening of the y domains.

**Figure 8:**
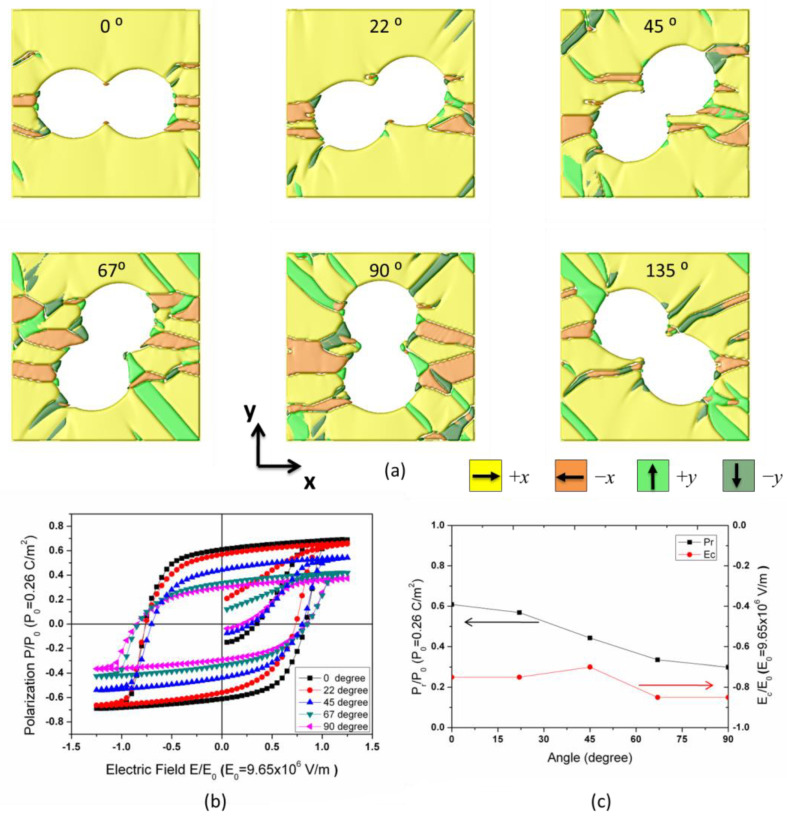
(**a**) Simulated ferroelectric domain structure for porous BTO with two connected circle-shaped pores, the angle between the line that connects the center of the two circles and the *x*-axis is 0°, 22°, 45°, 67°, 90°, and 135°, respectively. (**b**) Simulated *P*-*E* hysteresis loops for porous ferroelectrics are plotted in (**a**). Note that the volume fraction of the pores remains the same. (**c**) The variation of remanent polarization and coercive field with the angle.

In geometry, the ellipse-shaped pores have an analogous structure to connected circle-shaped pores, which inspired us to replace one elongated ellipse-shaped pore with two or more connected equal-axis pores. The latter can be fabricated in experiments using unidirectional freeze casting technology [26]. Figure 8a illustrates simulated porous samples with two connected circle-shaped pores of the same size instead of ellipse-shaped pores at a similar porosity level. These poled porous BTO materials exhibit similar ferroelectric properties during the switching process. Compared to ellipse-shaped pores, sharp tips at the connecting point between two circles seem intriguing; these tips efficiently prevent the formation of *y* domains. Consequently, the volume fraction of the stripe-like *y* domain region is lower compared to the samples with ellipse-shaped pores. The enhancement of *P*_r_ and *E*_c_ was demonstrated in the hysteresis loop and the variation plot of *P*_r_/*E*_c_ in Figure 8b,c.

### 3.3. The Influence on the Dielectric and Piezoelectric Properties

For design consideration, the variable value of dielectric constant *ε*_33_ and piezoelectric coefficient *d*_33_ enable a wide selection of dielectric and piezoelectric parameters. The two coefficient values can be estimated from the hysteresis loop of the porous ferroelectric body. For multi-domain structures in phase field simulation, the dielectric constant can be calculated from the change in polarization at the maximum applied electric field, according to *ε*_33_ = *ε*_11_ = (Δ$\bar{P_{1}}$)/*E*_max_. Similarly, the piezoelectric constant *d*_33_ can be calculated from the change in the average strain: *d*_33_ *= d*_11_ *=* (Δ$\bar{e_{1}}$)/*E*_max_ = *Q*_11_ × *P*_1_^2^ + *Q*_12_(*P*_2_^2^ + *P*_3_^2^)/*E*_max_.

**Figure 9:**
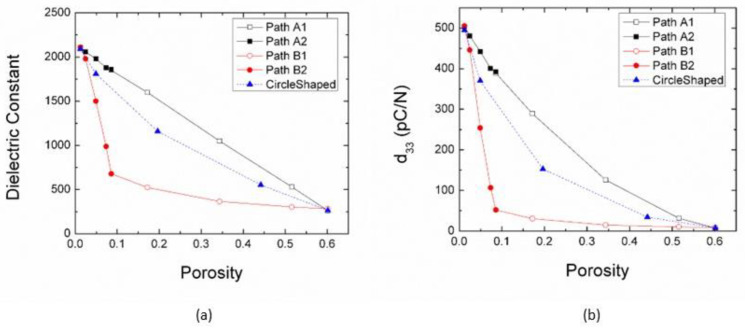
Dielectric and Piezoelectric properties for porous ferroelectric structures. (**a**) Variation of dielectric constant with porosity; (**b**) Piezoelectric coefficient *d*_33_ versus porosity at different porosity levels.

Both the dielectric and piezoelectric properties obtained in this simulation show a strong dependence on the porosity of materials, as shown in Figure 9a,b. The values for the calculated dielectric constant *ε*_33_ decreased from 2090 to 265 (by ~87%). The calculated *d*_33_ values decreased from 495 to 7.8 *p*C/N (by ~98%). There was a strong linear dependence of *ε*_33_ and *d*_33_ with porosity following path A. By contrast, *ε*_33_ and *d*_33_ decreased rather rapidly with porosity along path B-2, and the calculated dielectric constant *ε*_33_ decreased from 2090 to 679 (by ~67%), and the calculated *d*_33_ values decreased from 495 to 52.1 *p*C/N (by ~89%), then the *ε*_33_ and *d*_33_ slowly reach the minimum value along path B-1. The value of *ε*_33_ and *d*_33_ following a circle-shaped path lies between Path A and Path B. The formation of nano domain structures in this work leads to a predicted enhanced *d*_33_, which is higher than that of bulk materials. This phenomenon is consistent with experimental results reported by Shen et al. [45]. They found that the piezoelectric constant shows great dependence on the size of ferroelectric domains, with a maximum value of 416 *p*C/N observed with nanometer-sized domains.

These simulation results suggest that to enhance dielectric/piezoelectric properties for porous ferroelectrics, we should consider two contributions: the effect of porosity and the effect of domain structure. The reduction of the dielectric/piezoelectric properties of the pores is inevitable for porous materials with a certain porosity. To minimize the influence of the domain structure, we should align the major axis of ellipse-shaped pores or connected circle-shaped pores along the electric field direction; thus, we can expect the enhancement of piezoelectric properties. In experimental studies, there are several methods to control the direction of the porosity. For example, Zhang et al. employed removable fiber materials to prepare directionally arrayed pores in ceramics [46]. Additionally, Bowen and Zhang et al. [8,26] used the directional freeze casting method to create directional pores in various ferroelectric systems, such as Pb(Zr,Ti)O_3_ and BCZT (0.5Ba(Ca_0.8_Zr_0.2_)O_3_-0.5(Ba_0.7_Ca_0.3_)TiO_3_). Recently, template-assisted ion beam etching has been used to produce nanoporous arrays [47]. The shape and size of the pores can be controlled by the template. Additionally, pore concentration and external stress/strain effects are additional parameters that could impact the physical properties of porous ferroelectrics. These aspects are currently under investigation and will be addressed in future studies.

## 4. Summary

Using a phase model, we have shown the ferroelectric domain structures and their hysteresis loops with various circle-/ellipse-shaped pores in barium titanate. We designed three paths to demonstrate ferroelectric domain structure evolution with the pore size in radius by 8–56 nm and mapped key parameters for the hysteresis loop to a *P*_r_-*E*_c_ diagram. We found that the shape of the pores is a key point to achieve high ferroelectric/dielectric/piezoelectric properties in porous nanostructured ferroelectrics. The elastic field rebuilds the ferroelectric domain structure around the pores, and this domain structure, combined with the existing elastic field, further affects the hysteresis of the porous ferroelectrics. Three approaches are suggested to design the hysteresis loops: (i) through the shape control; (ii) rotate the ellipse-shaped pore or the external electric field; (iii) connect existing circle-shaped pores to create a similar ellipse-shaped structure. The dielectric constant *ε*_33_ and piezoelectric coefficient *d*_33_ are estimated in this work. Our simulation results indicate that aligning the major axis of ellipse-shaped pores or connecting circle-shaped pores along the direction of the electric field can minimize the influence of the domain structure and enhance the dielectric/piezoelectric properties. Furthermore, with the present work, the physical properties of porous ferroelectrics can be designed to suit lightweight/energy harvest applications. The obtained results point to a new direction for possible enhancements of nanostructured ferroelectric/dielectric/piezoelectric devices.

## Data Availability

The original contributions presented in the study are included in the article, further inquiries can be directed to the corresponding authors.

## References

[1] Khachaturyan R., Zhukov S., Schultheiß J., Galassi C., Reimuth C., Koruza J., von Seggern H., Genenko Y.A. (2017). Polarization-switching dynamics in bulk ferroelectrics with isometric and oriented anisometric pores. J. Phys. D Appl. Phys..

[2] Curecheriu L., Lukacs V.A., Padurariu L., Stoian G., Ciomaga C.E. (2020). Effect of porosity on functional properties of lead-free piezoelectric BaZr_0.15_Ti_0.85_O_3_ porous ceramics. Materials.

[3] Martínez-Ayuso G., Friswell M.I., Haddad Khodaparast H., Roscow J.I., Bowen C.R. (2019). Electric field distribution in porous piezoelectric materials during polarization. Acta Mater..

[4] Padurariu C., Padurariu L., Curecheriu L., Ciomaga C., Horchidan N., Galassi C., Mitoseriu L. (2017). Role of the pore interconnectivity on the dielectric, switching and tunability properties of PZTN ceramics. Ceram. Int..

[5] Zhu S., Cao L., Xiong Z., Lu C., Gao Z. (2018). Enhanced piezoelectric properties of 3-1 type porous 0.94Bi_0.5_Na_0.5_TiO_3_-0.06BaTiO_3_ ferroelectric ceramics. J. Eur. Ceram. Soc..

[6] Yap E.W., Glaum J., Oddershede J., Daniels J.E. (2018). Effect of porosity on the ferroelectric and piezoelectric properties of (Ba_0.85_Ca_0.15_)(Zr_0.1_Ti_0.9_)O_3_ piezoelectric ceramics. Scr. Mater..

[7] Gheorghiu F., Padurariu L., Airimioaei M., Curecheriu L., Ciomaga C., Padurariu C., Galassi C., Mitoseriu L. (2017). Porosity-dependent properties of Nb-doped Pb(Zr,Ti)O_3_ ceramics. J. Am. Ceram. Soc..

[8] Zhang Y., Roscow J., Lewis R., Khanbareh H., Topolov V.Y., Xie M., Bowen C.R. (2018). Understanding the effect of porosity on the polarisation-field response of ferroelectric materials. Acta Mater..

[9] Yan M., Xiao Z., Ye J., Yuan X., Li Z., Bowen C., Zhang Y., Zhang D. (2021). Porous ferroelectric materials for energy technologies: Current status and future perspectives. Energy Environ. Sci..

[10] Zhang Y., Bowen C.R., Ghosh S.K., Mandal D., Khanbareh H., Arafa M., Wan C. (2019). Ferroelectret materials and devices for energy harvesting applications. Nano Energy.

[11] Chen Y., Wang N., Ola O., Xia Y., Zhu Y. (2021). Porous ceramics: Light in weight but heavy in energy and environment technologies. Mater. Sci. Eng. R Rep..

[12] Xu T., Wang C.-A. (2016). Control of pore size and wall thickness of 3-1 type porous PZT ceramics during freeze-casting process. Mater. Des..

[13] Mercadelli E., Galassi C. (2021). How to Make Porous Piezoelectrics? Review on Processing Strategies. IEEE Trans. Ultrason. Ferroelectr. Freq. Control..

[14] Guo J., Wu Q., Zhang C., Li Y., Nie M., Wang Q., Liu Y. (2022). Porosity manipulation to boost piezoelectric output via supercritical carbon dioxide foaming and surface modification. Mater. Des..

[15] Wang J., Wylie-van Eerd B., Sluka T., Sandu C., Cantoni M., Wei X.K., Kvasov A., McGilly L.J., Gemeiner P., Dkhil B. (2015). Negative-pressure-induced enhancement in a freestanding ferroelectric. Nat. Mater..

[16] Kvasov A., McGilly L.J., Wang J., Shi Z., Sandu C.S., Sluka T., Tagantsev A.K., Setter N. (2016). Piezoelectric enhancement under negative pressure. Nat. Commun..

[17] Ferreira P., Hou R.Z., Wu A., Willinger M.G., Vilarinho P.M., Mosa J., Laberty-Robert C., Boissière C., Grosso D., Sanchez C. (2012). Nanoporous piezo- and ferroelectric thin films. Langmuir.

[18] Matavž A., Bradeško A., Rojac T., Malič B., Bobnar V. (2019). Self-assembled porous ferroelectric thin films with a greatly enhanced piezoelectric response. Appl. Mater. Today.

[19] Castro A., Ferreira P., Rodriguez B.J., Vilarinho P.M. (2015). The role of nanoporosity on the local piezo and ferroelectric properties of lead titanate thin films. J. Mater. Chem. C.

[20] Chen F., Yang C., An Z., Zhang X., Zhou T., Chen N. (2022). Direct-ink-writing of multistage-pore structured energy collector with ultrahigh ceramic content and toughness. Mater. Des..

[21] Vorotyntsev D.A., Vishnevskiy A.S., Seregin D.S., Sigov A.S., Vorotilov K.A. (2025). Temperature evolution of pore structure in ferroelectric PZT films prepared by molecular self-assembly. J. Adv. Dielectr..

[22] Suzuki N., Osada M., Billah M., Alothman Z.A., Bando Y., Yamauchi Y., Hossain M.S. (2017). Origin of thermally stable ferroelectricity in a porous barium titanate thin film synthesized through block copolymer templating. APL Mater..

[23] Stancu V., Lisca M., Boerasu I., Pintilie L., Kosec M. (2007). Effects of porosity on ferroelectric properties of Pb(Zr_0.2_Ti_0.8_)O_3_ films. Thin Solid Film..

[24] Billah M., Terasawa Y., Masud M.K., Asahi T., Hegazy M.B.Z., Nagata T., Chikyow T., Uesugi F., Hossain M.S.A., Yamauchi Y. (2024). Giant piezoresponse in nanoporous (Ba,Ca)(Ti,Zr)O_3_ thin film. Chem. Sci..

[25] Delimova L., Seregin D., Orlov G., Zaitseva N., Gushchina E., Sigov A., Vorotilov K. (2023). Porous PZT films: How can we tune electrical properties?. Materials.

[26] Zhou X., Zhou K., Zhang D., Bowen C., Wang Q., Zhong J., Zhang Y. (2022). Perspective on porous piezoelectric ceramics to control internal stress. Nanoenergy Adv..

[27] Roscow J.I., Pearce H., Khanbareh H., Kar-Narayan S., Bowen C.R. (2019). Modified energy harvesting figures of merit for stress- and strain-driven piezoelectric systems. EPJ Spec. Top..

[28] Huang J., Tan P., Wang F., Li B. (2022). Ferroelectric memory based on topological domain structures: A phase field simulation. Crystals.

[29] Hou X., Li H., Shimada T., Kitamura T., Wang J. (2018). Effect of geometric configuration on the electrocaloric properties of nanoscale ferroelectric materials. J. Appl. Phys..

[30] Chen H., Hou X., Chen J., Chen S., Hu P., Wu H., Wang J., Zhu J. (2020). Large electrostrain induced by reversible domain switching in ordered ferroelectric nanostructures with optimized geometric configurations. Nanotechnology.

[31] Ma L.L., Ji Y., Chen W.J., Liu J.Y., Liu Y.L., Wang B., Zheng Y. (2018). Direct electrical switching of ferroelectric vortices by a sweeping biased tip. Acta Mater..

[32] Zhou M.-J., Yang T., Wang J.-J., Ren Z., Chen L.-Q., Nan C.-W. (2020). Nanopore-induced dielectric and piezoelectric enhancement in PbTiO$_3$ nanowires. Acta Mater..

[33] Wang J.-J., Wang B., Chen L.-Q. (2019). Understanding, predicting, and designing ferroelectric domain structures and switching guided by the phase-field method. Annu. Rev. Mater. Res..

[34] Zhao H., Wu P., Du L., Du H. (2018). Effect of the nanopore on ferroelectric domain structures and switching properties. Comput. Mater. Sci..

[35] Xie C., Zhao H., Du L., Du H., Wu P. (2021). Enhanced ferroelectricity for nanoporous barium titanate: A phase-field prediction. Philos. Mag. Lett..

[36] Van Lich L., Shimada T., Wang J., Kitamura T. (2017). Self-ordering of nontrivial topological polarization structures in nanoporous ferroelectrics. Nanoscale.

[37] Kargupta R., Venkatesh T. (2006). Electromechanical response of porous piezoelectric materials. Acta Mater..

[38] Li Y., Cross L., Chen L. (2005). A phenomenological thermodynamic potential for BaTiO_3_ single crystals. J. Appl. Phys..

[39] Merz W.J. (1954). Domain Formation and Domain Wall Motions in Ferroelectric BaTiO_3_ Single Crystals. Phys. Rev..

[40] Lines M.E., Glass A.M. (2001). Principles and Applications of Ferroelectrics and Related Materials.

[41] Geng L.D., Jin Y.M., Tan D.Q., Wang Y.U. (2018). Computational study of nonlinear dielectric composites with field-induced antiferroelectric-ferroelectric phase transition. J. Appl. Phys..

[42] Lukacs V.A., Stanculescu R., Curecheriu L., Ciomaga C.E., Horchidan N., Cioclea C., Mitoseriu L. (2020). Structural and Functional Properties of BaTiO_3_ Porous Ceramics Produced by Using Pollen as Sacrificial Template. Ceram. Int..

[43] Jo J.Y., Kim Y.S., Noh T.W., Yoon J.-G., Song T.K. (2006). Coercive Fields in Ultrathin BaTiO_3_ Capacitors. Appl. Phys. Lett..

[44] Sheeraz M., Tran V.D., Jo Y.J., Kim G., Cho S., Sohn C., Kim I.W., Shin Y.H., Ahn C.W., Kim T.H. (2024). Defect Engineering of Ferroelectric Hysteresis in Lead-Free Bi_1/2_(Na,K)_1/2_TiO_3_ Thin Films. ACS Appl. Electron. Mater..

[45] Shen Z.-Y., Li J.-F. (2010). Enhancement of Piezoelectric Constant d_33_ in BaTiO_3_ Ceramics due to Nano-Domain Structure. J. Ceram. Soc. Jpn..

[46] Zhang G.J., Yang J.F., Ohji T. (2001). Fabrication of porous ceramics with unidirectionally aligned continuous pores. J. Am. Ceram. Soc..

[47] Guo Y., Peng B., Qiu R., Dong G., Yao Y., Zhao Y., Zhou Z., Liu M. (2023). Self-Rolling-up Enabled Ultrahigh-Density Information Storage in Freestanding Single-Crystalline Ferroic Oxide Films. Adv. Funct. Mater..

